# Predictive model of positive attitudes in Spanish health care students towards older people: assessment and associated factors

**DOI:** 10.3389/fpsyt.2025.1436930

**Published:** 2025-02-03

**Authors:** Jesús González-Moreno, Ana Isabel Agustí, Javier Guillem-Saiz, María Antonia Parra-Rizo, María Cantero-García

**Affiliations:** ^1^ Universidad Internacional de Valencia, Valencia, Spain; ^2^ Miguel Hernández University of Elche, Elche, Spain; ^3^ Universidad a Distancia de Madrid, Madrid, Spain

**Keywords:** attitudes, older adults, aging, character strengths, heathcare

## Abstract

**Background:**

Attitudes influence how individuals interact toward older people, which in turn impacts the way people care for them. This is a key factor to consider for future health professionals who will frequently work with older individuals. This issue has been approached by simply quantifying these attitudes (negative, neutral, or positive). However, this work aims to not only quantify these attitudes but to study other associated variables and develop a predictive model of positive attitudes toward the older people in a sample of Spanish university students of health care careers.

**Methods:**

A total of 338 students were surveyed using a sociodemographic questionnaire and two validated scales: the Spanish versions of the Kogan Scale of Attitudes toward Older People (KAOP) and the Global Assessment of Character Strengths-24 (GACS-24). This study employed a quantitative, cross-sectional, ex post facto design, with data analyzed through correlation and regression techniques to identify predictors of positive attitudes toward older adults.

**Results:**

The results exhibited significantly positive attitudes toward older people. The analysis identified “love” (a character strength) and “interest in aging issues” as significant predictors of these attitudes.

**Conclusions:**

This study concludes that positive attitudes toward older adults are significantly associated with the character strength of ‘love’ and ‘interest in aging issues,’ highlighting the importance of these variables in shaping attitudes among future health professionals.

## Introduction

As societies continue to age, older adults are projected to make up an increasingly large proportion of the global population. This demographic shift underscores the need for better attention and care for older adults. However, societal prejudices and negative attitudes remain major barriers to providing high-quality care (1–3). Specifically, many older adults are perceived as dependent, frail, or resistant to new experiences, which can lead to suboptimal care and hinder their social participation. Healthcare professionals, influenced by such stereotypes, may underestimate older patients’ capacity to benefit from certain treatments, while broader societal attitudes can reduce support and inclusion for older individuals. Consequently, these biases limit opportunities for older adults to engage fully in community life, potentially affecting both their physical and emotional well-being. Addressing these prejudices is therefore crucial to ensuring not only that older adults receive comprehensive, person-centered care, but also that they remain integrated and valued within society.

Positive attitudes toward older adults greatly influence their integration, participation, and overall well-being. Studies have demonstrated that fostering psychological strengths such as hope and gratitude can enhance both quality of life and quality of care for this population (4, 5). For example, the positive psychology movement emphasizes focusing on strengths and capacities, which are widely acknowledged as contributors to well-being (4). Furthermore, cultural factors can also shape perceptions of aging; for instance, research in Indigenous communities in Northern Ontario revealed more favorable attitudes toward older adults compared to other groups, highlighting the powerful role of culture in shaping attitudes (6). Similarly, interventions centered on psychological constructs such as belief in a just world and a strong sense of coherence have been linked to more positive attitudes toward aging, thereby promoting better integration and care (7, 8).

During the COVID-19 pandemic, societal responses further underscored the importance of positive attitudes. Supportive public measures affirmed the value of older adults, enhancing their mental and physical health, whereas negative reactions exacerbated ageism and related health problems (9). Currently, aging is still primarily viewed through a lens of negative stereotypes, depicting older adults as passive, slow, melancholy, or frequently dependent and cognitively impaired (10). These stereotypes not only impede older adults’ social integration but also adversely affect the quality of care they receive.

In Spain, societal attitudes toward older adults are shaped by a mix of traditional family values and emerging ageist narratives. While older adults are often respected as key figures within the family, stereotypes of dependency and frailty persist, influencing social and professional interactions (11, 12). Moreover, studies indicate that older adults are underrepresented in Spanish media, where coverage tends to emphasize health-related challenges rather than active or positive roles, thus reinforcing limited views of aging (13).

Ageism refers to stereotypes and prejudices based on age, which may or may not lead to discriminatory actions or policies. In contrast, age discrimination specifically denotes biased actions or regulations that negatively affect older adults. Both phenomena have serious implications in healthcare (14). Research shows that age significantly influences how medical treatments are allocated (15). Negative attitudes toward older adults can result in suboptimal care, as their recovery potential is often underestimated, and they may be denied essential or innovative treatments. Additionally, ageism can lead to misdiagnoses, wherein medical conditions are incorrectly attributed solely to advanced age. However, this discrimination can be mitigated through inclusive policies, educational programs aimed at dispelling aging myths and promoting empathy, and intergenerational interventions that foster mutual understanding and respect (14).

In this context, improving care for older adults requires attention to the attitudes of healthcare professionals, particularly students who will soon serve this growing population (16–18). These attitudes—mental or behavioral tendencies—influence how care is provided and are open to change (19–22). They can evolve through social or personal factors, pointing to the potential of targeted educational programs to foster more positive attitudes (19, 23). Research suggests that improving these attitudes can enhance the quality of care for older adults (24). Conversely, negative attitudes may result in inadequate care (21, 25). Therefore, focusing on attitudinal development during academic training is essential to ensure that future healthcare professionals are prepared to provide high-quality care for older adults (26).

From a theoretical standpoint, several frameworks shed light on how and why such attitudes form and may be altered. Social Identity Theory (27) suggests that individuals categorize themselves and others into social groups, leading to in-group favoritism and out-group biases, which can include negative stereotypes of older adults. The Contact Hypothesis (28) proposes that meaningful, equal-status interactions with older adults, supported by institutional norms, can reduce prejudice. In healthcare contexts, these frameworks highlight the potential impact of structured educational experiences that encourage empathy, a critical mechanism according to the Empathy–Altruism Hypothesis (29). Additionally, the Theory of Planned Behavior (30) underscores how attitudes, social norms, and perceived behavioral control shape intentions and behaviors; this framework may help explain how training interventions affect students’ readiness to care for older adults.

Studies indicate that health science students’ positive attitudes can be linked to factors such as age, gender, and other sociodemographic variables (31, 32), as well as to certain personal traits. These traits, defined as internal, positive, and stable components of an individual’s personality (33), are crucial for coping with life’s challenges, relating to others, and seeking both personal and community well-being. Examples of such strengths include kindness, gratitude, humility, love, perseverance, and honesty. They represent core elements of an individual’s character that shape how people behave and react, especially in interpersonal contexts (34). These qualities, which encompass the ability to empathize and navigate social interactions effectively, are essential to forming positive attitudes toward older adults. Moreover, these skills can be cultivated and expanded (35–37). A previous study (38) found that positive attitudes toward older adults correlate with personal variables such as the capacity to give and receive love, kindness, gratitude, and humility. The positive psychology movement further highlights the value of emphasizing strengths and abilities, including hope and gratitude, which are universally recognized as contributors to well-being (4). Additionally, constructs like the belief in a just world and a strong sense of coherence have been linked to more positive attitudes toward aging, suggesting that psychological well-being significantly influences perceptions of older adults (7). Despite their importance, however, relatively few studies have examined how these personal variables impact attitudes toward older people, emphasizing the need for additional research in this field. In conclusion, improving the care of older adults may hinge on understanding and strengthening the positive attitudes of future healthcare professionals. The present study aims to analyze health students’ attitudes toward older adults, as well as the sociodemographic and personal variables or strengths associated with these attitudes. By developing a predictive model, we may be able to identify key variables to target in future intervention programs, thereby ultimately enhancing care for older adults.

Previous research suggests that students in health-related fields often demonstrate relatively positive attitudes toward older adults, partly due to their academic exposure to topics related to aging. Still, these attitudes are not uniformly strong, indicating opportunities for targeted improvement (39). We hypothesize that attitudes may vary based on sociodemographic factors such as age and gender (with gender treated as a categorical variable). Additionally, personal variables—such as humility, love, kindness, and gratitude—are expected to correlate with more positive attitudes, consistent with existing literature. We further propose that combining sociodemographic and personal variables will provide a more precise prediction of positive attitudes toward older adults among health science students, suggesting potential pathways for educational and professional interventions.

## Materials and methods

### Study design and population

This quantitative study employed an ex post facto design with a cross-sectional approach, involving 338 university students enrolled in health-related programs. The inclusion criteria required participants to be actively pursuing studies in health-related programs at three Spanish universities. Participants were recruited indirectly through social media and emails disseminated by the participating universities. The sample consisted of 86.1% women, 13.6% men, and 0.3% who either chose not to specify or selected another category. Ages ranged from 18 to 65 years (M = 30.01; *SD* = 9.80), reflecting the demographic diversity of university students in health-related programs in Spain, where mature students are common. Additionally, 73.4% of the participants reported a medium income level.

The sampling method used was snowball sampling, which does not allow for precise tracking of the total number of individuals invited to participate. Of those who received the survey, only one person declined to provide informed consent, and no incomplete questionnaires were submitted. This resulted in a final sample of 338 participants.

### Instruments

A sociodemographic questionnaire was used to collect key information about participants such as gender and age, and to better understand the context of participants in relation to their experience and attitudes towards aging. This questionnaire included questions about interactions with older adults, as well as interest and willingness to learn about aging-related topics, crucial elements for assessing predisposition towards geriatric care (see **Table 1**).

**Table 1:** General and specific sociodemographic variables.

Variable	Description/Response Options
Gender	1 = Men, 2 = Women
Age (years)	Chronological age in years
Older adults in the family	“Is there anyone in your family over 65 years of age?” (1 = Yes, 2 = No)
Living with older adults	“Have you ever lived with people over 65?” (1 = Yes, 2 = No)
Interest in aging issues	“Are you interested in topics related to old age, ageing, or gerontology?”
	Response options: a lot, somewhat, a little, very little, none
Desire to learn about aging	“Would you like to learn about topics related to old age, ageing, or gerontology?”
	Response options: a lot, somewhat, a little, very little, none
Years of study experience	Number of years studying topics related to aging
Years of work experience	Number of years working in the field of aging
Years of volunteer experience	Number of years volunteering in the field of aging
Years of informal care experience	Number of years providing informal care to older adults

The questionnaire confirmed that participants were enrolled in healthcare programs; however, no data were collected regarding their specific field of study or university affiliation. This approach was adopted to ensure anonymity and prevent potential stigmatization, in accordance with the ethics committee’s requirements.

Additionally, two standardized instruments were employed. First, the Spanish version of the Kogan Scale of Attitudes toward Older People (KAOP) was used to assess attitudes toward older adults. The KAOP, validated for Spanish-speaking populations (40), comprises 34 statements—some positively and some negatively worded—rated on a 6-point Likert scale ranging from “strongly disagree” to “strongly agree.” Higher scores indicate more positive attitudes toward older people, with negatively worded items reverse-coded to calculate a total score. The maximum possible score is 204, while scores around 102 are considered neutral; scores below 102 are deemed negative, and those above 102 are regarded as positive (41, 42). Example items include “Older people should have more power in society” and “Older people are irritable, grumpy, and unpleasant.” Previous validation research reported a reliability of α = .82 (40). This instrument has been widely used in current research (42) and was also applied in another published study (43).

Second, the Spanish version of the Global Assessment of Character Strengths-24 (GACS-24) (44) was administered to evaluate personal character traits. This tool consists of 24 items rated on a 7-point Likert scale from 1 (“strongly disagree”) to 7 (“strongly agree”). Example items include “You are considered a creative person; you see, do, and/or create things that are useful; you come up with unique ways to solve problems and be productive,” and “You are analytical; you examine things from all points of view; you do not jump to conclusions but try to weigh all the evidence when making decisions.” The questionnaire assesses how essential, natural, effortless, uplifting, and energizing each strength feels to the respondent. Scores for each strength are calculated by averaging the responses on its relevant items. The mean item reliability has been reported at α = .78 (45). This instrument has also been employed in previously published research (46).

In the current study, the Spanish version of the KAOP demonstrated high reliability (α = .85), indicating a strong internal consistency among participant responses. Similarly, the Spanish version of the GACS-24 achieved a Cronbach’s alpha of.81 in this sample, confirming its reliability within our research context.

### Study procedures

An online survey containing all sociodemographic variables and instruments was created using Qualtrics software, which provides robust data protection. An anonymous link was then generated and distributed to multiple Spanish universities, where it was shared with health sciences students via mailing lists and institutional websites. Data were collected in November and December 2021. At the beginning of the survey, participants were informed about the objectives of the study and were asked to provide informed consent.

This study adhered to the ethical principles set forth in the Declaration of Helsinki. It was approved by the Human Research Ethics Committee (CEISH) of the International University of Valencia on September 23, 2021 (Reference: CEID2021_12). Explicit consent to participate was obtained from all study subjects.

### Data analysis

Data analysis was performed using SPSS Version 25. First, a descriptive analysis of the variables under investigation was conducted. Next, correlations between these variables and participants’ positive attitudes toward older adults were examined. Finally, regression analyses were carried out using the forward selection method (a stepwise procedure that tests entries based on the significance of the score statistic and tests removals based on the probability of a likelihood ratio statistic, drawing on conditional parameter estimates) to identify independent predictors of positive attitudes toward older adults. For this regression analysis, only sociodemographic and character strength variables that showed a significant correlation with the attitude score were included.

Before conducting the analyses, all necessary assumptions (normality, linearity, homoscedasticity, and diagnostic tests for multicollinearity and independence of errors) were verified. Statistical significance was set at p < 0.05, and all tests were two-tailed.

## Results

### Exploration of the variables studied in the sample and their relationship with attitudes toward older adults

#### Attitude scores and gender differences

First, the mean attitude score toward older adults was 146.26 (*SD* = 16.78), ranging from 91 to 190. No significant differences emerged between women (*M* = 147.10, *SD* = 16.93) and men (*M* = 141.38, *SD* = 15.17) regarding this attitude score, *t*(250) = −1.92, *p* = .55. This finding suggests that gender does not influence attitudes toward older adults.

#### Descriptive analyses and character strengths

**Tables 2** present the exploratory analyses of the variables, including descriptive results and their relationship with positive attitudes toward older adults. The majority of respondents had at least one family member over the age of 65, although fewer had lived with an older adult. Most participants’ experience with older adults came from informal caregiving. In terms of interest in aging-related topics, 88.9% reported being somewhat or very interested, while 76.3% indicated being somewhat or very interested in receiving formal training in this area. With respect to character strengths, the traits most commonly associated with older adults by participants were kindness, love of learning, and gratitude.

**Table 2:** Descriptive statistics and correlation of interval variables with attitudes toward older people score.

Variable	*Mean (SD)*	Range	Pearson Correlation with Attitude
Age	30 (9.8)	47	.109
Interest in aging issues	3.45 (1.2)	5	.132*
Desire to learn about aging	3.78 (1.05)	5	.018
Years of experience in studies related to the field of old age	2.41 (3.92)	45	-.092
Years of experience working in the field of old age	2.49 (3.41)	15	-.013
Years of experience volunteering in the field of old age	0.98 (1.84)	11	-.016
Years of experience in informal care in the field of old age	3.76 (4.34)	29	.056
Creativity	5.37 (1.31)	5	-.005
Curiosity	5.81 (1.14)	6	.116
Judgement	5.75 (1.22)	6	.043
Love for learning	6.04 (1.11)	6	.121
Perspective	5.74 (1.15)	6	.110
Bravery	5.36 (1.40)	6	.073
Perseverance	5.75 (1.23)	6	.075
Honesty	5.99 (1.09)	6	.090
Zest	5.25 (1.46)	6	.020
Love	5.64 (1.45)	6	.253**
Kindness	6.12 (1.00)	6	.099
Social intelligence	5.66 (1.22)	6	.178**
Teamwork	5.77 (1.10)	6	-.041
Fairness	5.74 (1.24)	6	.131*
Leadership	5 (1.52)	6	.023
Forgiveness	5.04 (1.52)	6	.117
Humility	5.74 (1.2)	6	.084
Prudence	5.31 (1.38)	6	.018
Self-regulation	5.05 (1.42)	6	.036
Appreciation for beauty	5.58 (1.30)	6	-.020
Gratitude	5.97 (1.22)	6	.114
Hope	5.35 (1.44)	6	-.027
Humor	5.55 (1.30)	6	.002
Spirituality	4.69 (1.92)	6	-.045

*p < 0.05, **p < 0.01.

Second, some variables demonstrated a significant association with positive attitudes toward older adults. These included “interest in aging issues” and the character strengths of love, fairness, and social intelligence.

Although the age distribution of the sample leaned toward younger participants, additional checks confirmed that this skewness did not violate the assumptions necessary for regression analysis. Future studies may benefit from stratified sampling or alternative statistical approaches to investigate potential non-linear relationships with age.

### Results of the predictive model of positive attitudes toward older people

A multiple regression analysis was conducted using the forward selection method to predict the effect of four variables (“love,” “interest in aging issues,” “social intelligence,” and “fairness”) on the attitudes of university students in the health care field toward older adults. The variables included in the regression analysis were pre-selected based on their significant bivariate correlations with attitudes toward older adults, ensuring their theoretical and statistical relevance. The forward selection method sequentially included predictors in the model based on their statistical contribution, retaining only those that significantly improved the model’s fit (*p* < 05). The final model retained two significant predictors: “love” and “interest in aging issues. The regression equation was statistically significant *F*(2, 249) = 13.06, *p* = <.001, β-1 = .99. The R^2^ value was.10, which indicates that 10% of the variance in the attitude score can be explained by the model. The regression equation was 119.1 + 2.48* (“love” score) + 3.86*(“interest in aging issues”), the score for attitudes toward older adults increased by 2.48 points for each point of increase in the variable “love” and 3.86 for each point of increase in “interest in aging issues”. **Table 3** that will show the findings of the regression analysis.

**Table 3:** Regression analysis predicting positive attitudes toward older people.

Predictor	B (Unstandardized)	SE	t-value	p-value	95% CI
Love	0.45	0.08	5.62	<0.001	[0.29, 0.61]
Interest in Aging Issues	0.32	0.07	4.57	<0.001	[0.18, 0.46]
Social Intelligence	0.05	0.06	0.83	0.41	[-0.06, 0.16]
Fairness	-0.03	0.05	-0.60	0.55	[-0.13, 0.07]

Model Summary: • R^2^ = 0.10 • Adjusted R² = 0.09 • *F*(2, 335) = 12.15, *p* < 0.001

Note: • B = Unstandardized regression coefficient • SE = Standard Error • CI = Confidence Interval

## Discussion

This study aimed to evaluate the attitudes of university students in healthcare fields (future healthcare professionals) toward older adults and to identify variables associated with positive attitudes that could inform the development of a predictive model. Attitudes are critical in shaping how individuals interact with others and in determining the quality of care provided to older adults. Our findings align with existing literature emphasizing the role of positive attitudes in improving care outcomes for this population (47). For example, evidence-based programs like Nurses Improving Care for Healthsystem Elders (NICHE) integrate geriatric knowledge into healthcare settings, thereby enhancing patient outcomes (48). By contrast, negative attitudes rooted in stereotypes and limited geriatric training can lead to suboptimal care—particularly in high-stress environments such as intensive care units (49).

The results indicate that, on average, healthcare students hold positive attitudes toward older adults, which is consistent with previous research among healthcare students and professionals (50–52). However, the scores also suggest room for improvement, highlighting the need for further research on strategies to enhance attitudes toward older adults and, ultimately, the quality of care they receive.

### Sociodemographic factors

Debate continues regarding whether sociodemographic variables significantly influence positive attitudes toward older adults. While some studies have found significant associations (52), others have not (53). In our study, variables such as gender, living with older adults, and experience with older adults did not significantly correlate with positive attitudes. Given the predominantly young age of our sample, one possible explanation is that respect for older adults may be transmitted intergenerationally, regardless of gender (54), suggesting that cultural or educational interventions might shift these attitudes. Although previous research links living with and caring for older adults with more positive attitudes (55, 56), our study did not replicate these findings. This result is especially notable given the high proportion of participants who lived with or had relatives over 65, reflecting Spain’s cultural context (52). Since mere contact with older adults does not appear sufficient to alter attitudes, further investigation into the nature and quality of interactions is warranted.

### Interest in aging-related topics

Our findings suggest a high level of interest in aging-related topics among participants, likely reflecting the growing multidisciplinary focus on aging (51). Previous studies indicate that gerontological education correlates with more positive attitudes toward older adults (57, 58). In this research, a greater interest in aging was associated with more positive attitudes. Nevertheless, interest alone does not necessarily translate into a desire for formal education in the field. Hence, nurturing students’ curiosity and providing structured educational pathways may be crucial for enhancing attitudes in practical settings.

### Character strengths

This study identified correlations between specific character strengths—particularly fairness, social intelligence, and love—and positive attitudes, supporting the notion that personal factors are integral to forming various attitudes. Love appears to exert the greatest influence on positive attitudes (18), possibly reflecting an inherent predisposition toward empathy and supportive caregiving. Empathy has previously been shown to enhance attitudes toward older adults (25, 59, 60). However, we did not replicate findings from other studies that highlight gratitude and hope (39), suggesting that additional research is needed to clarify these relationships. Given the lack of extensive studies on these variables, our findings contribute novel insights into their potential importance.

### Predictive model

In sum, while multiple variables correlate with more positive attitudes toward older adults, “interest in aging issues” and the character strength “love” emerged as significant predictors. Notably, the model explained 10% of the variance, which is acceptable within psychological research (61). Despite the modest proportion of explained variance, the model’s parsimony and significance underscore the practical value of these predictors. The selective inclusion of “love” and “interest in aging issues” was guided by both conceptual relevance and statistical robustness, avoiding overfitting and preserving interpretability. Future studies using larger samples could incorporate additional predictors to expand on these findings.

### Ageism and discrimination

The link between ageism and discrimination against older individuals, encompassing both physical and psychological risks, is well-documented. However, evidence regarding effective interventions to reduce such discrimination remains limited. A systematic review suggests that simple, cost-effective interventions incorporating educational elements and intergenerational contact may be foundational in reducing age-based discrimination (62). This insight paves the way for future research initiatives.

### Broader context in Spain

Finally, our findings resonate with broader societal trends in Spain, where respect for older generations coexists with persistent ageist attitudes. Surveys show that while many Spaniards recognize the value of older adults, nearly 40% identify age discrimination as a major concern in professional environments (11, 12). Media portrayals often highlight dependency and vulnerability, perpetuating stereotypes that can negatively influence care quality (13). These trends mirror similar dynamics in other Latin and Mediterranean countries, which rely heavily on family-centered care. In contrast, Northern European countries typically emphasize institutionalized care systems, driven by societal values of independence and public responsibility. Considering these cultural factors is essential for designing effective, context-specific interventions.

### Limitations

This study relied on a sample of university students in healthcare, which may not be representative of the broader population. Additionally, we did not differentiate among specific future professions (e.g., medicine, nursing), and our sample showed a pronounced female majority, which could have shaped the findings. Although the age distribution reflects the reality of health science programs in Spain, it skews toward younger individuals, limiting generalizability. Future research should consider stratified sampling or further analyses focusing on age. The relatively low percentage of explained variance points to the need for examining other potential correlates. Finally, the cross-sectional, correlational design precludes causal conclusions, highlighting the importance of longitudinal or experimental research to clarify the direction of these relationships.

## Social and practical implications

The findings have significant implications for healthcare education and practice. With an aging population and a pressing need for high-quality care, promoting positive attitudes among future healthcare professionals is crucial. Our results highlight the value of gerontological education and the character strength “love” in fostering empathy and attachment, both critical for high-quality care. Incorporating these elements into healthcare curricula could not only improve clinical outcomes but also combat age-based discrimination. Moreover, these results are relevant beyond Spain; although the strong family-centered culture is characteristic of Mediterranean societies, similar principles may apply globally, warranting cross-cultural comparisons.

In conclusion, while future healthcare professionals generally demonstrate positive attitudes toward older adults, there is scope for improvement. Interest in aging issues and the character strength “love” emerged as key predictors of more favorable attitudes, providing practical points of intervention to enhance care and well-being for older adults. Further research is needed to explore additional predictors and to validate targeted interventions, ideally involving not only students but also practicing professionals and other demographic groups. Such endeavors will contribute significantly to improving the quality of life and care for the rapidly growing older population.

## Data Availability

The raw data supporting the conclusions of this article will be made available by the authors, without undue reservation.
